# Ultraviolet radiation rate in Mashhad, Iran

**DOI:** 10.1016/j.dib.2018.05.116

**Published:** 2018-05-25

**Authors:** Maliheh Akhlaghi, Majid Radfard, Hossein Arfaeinia, Marzieh Soleimani, Adibeh Fallahi

**Affiliations:** a Students Research Committee, Gonabad University of Medical Sciences, Gonabad, Iran; b Department of Biostatistics, Torbat Heydariyeh University of Medical Sciences, Torbat Heydariyeh, Iran; c Student׳s Scientific Research Center, Tehran University of Medical Sciences, Tehran, Iran; d Department of Environmental Health Engineering, School of Public Health, Bushehr University of Medical Sciences, Bushehr, Iran; e Students Research Committee, Kermanshah University of Medical Sciences, Kermanshah, Iran

**Keywords:** Radiation, Ultraviolet, Ionizing radiation, Mashhad

## Abstract

Todays, Climate change can be effect on the intensity of ultraviolet (UV) radiation and cause of many human diseases. In this cross-sectional study, changes of the intensity of UV ray were associated with the changes in latitude and longitude, height, climatic conditions, natural and human-made artifacts. Given that the highest radiation intensity was at the beginning of the summer, the radiation rate of UV ray in Mashhad was measured in the summer using a Hagner radiometer, the UV-A model. The radiation rate of the UV ray was determined in 2000 stations, which were 5 km far from each other. Data were analyzed using SPSSv16 software, T-test, and ANOVA tests. The results of this study showed that the radiation rate of UV ray in Mashhad was 0.49±0.143 mSv per year. The findings showed that latitudinal and longitudinal changes did not have a significant effect on the intensity of UV radiation (P > 0.001). The changes in the height above the sea level influenced the irradiance rate of UV and increasing the height above the sea level raised UV radiation (P < 0.001). Human artifacts significantly changed the rate of UV radiation (P < 0.001). Cloudy, semi-cloudy and sunny conditions had the most effects on UV radiation (P < 0.001). The results revealed that the average rate of UV ray in Mashhad was below the global standard (10 W/m^2^ for UV ray), and traffic in open air could not be risky.

**Specifications Table**

**Table:** 

Subject area	Environmental sciences
More specific subject area	Description of hazardous natural rays
Type of data	Tables and figures
How data was acquired	The data were collected by 2000 sampling stations in different parts of the city. The UV radiation was measured using Hagner radiometer (model UV-A in watt/m2).
Data format	Raw, analyzed
Experimental factors	The sampling points were approximately 5000 meters far from each other
Experimental features	In this study, all conditions for the selection of sampling points, as well as the method for measuring ultraviolet radiation, were in accordance with the conditions set out in the valid references.
Data source location	Mashhad, Khorasan Razavi province, Iran
Data accessibility	Data are included in this article

**Value of the data**

• UV is the most important cosmic rays. UV radiation with medium and short wavelengths endangers human and living organisms in the open air. Therefore, continuous and periodic monitoring of this radiations is necessary. The data of this study is aimed at achieving the mentioned goal.
• Due to, no study has been done on this topic in the region so far. The data of this study can help to better understand the rate of hazardous Ultraviolet ray in the area and provide further studies.
• According to The data of this study, the average of UV ray in the Mashhad city was safe and below the global standard (10 W/m^2^ for UV ray)

## 1 Data

### 1.1 Relationship between longitudinal changes and the intensity of UV radiation in Mashhad

**Fig. 1:**
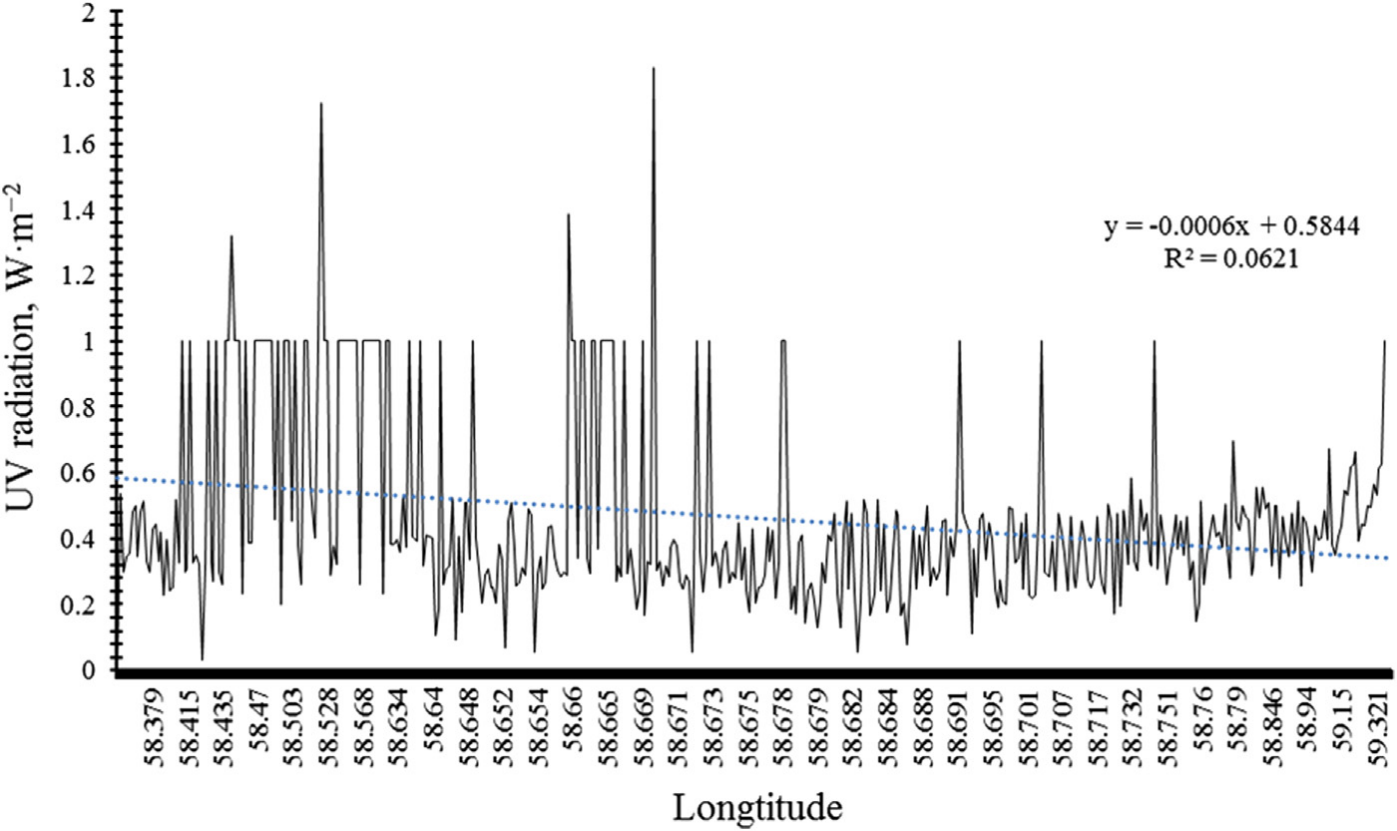
Comparison of the relationship between longitude and UV.

Fig. 1 show the relationship between changes in longitude and UV in Mashhad. These figures show that longitudinal changes have no effect on the intensity of UV radiation (P-value > 0.001).

### 1.2 Relationship between latitudinal changes and the intensity of UV radiation in Mashhad

**Fig. 2:**
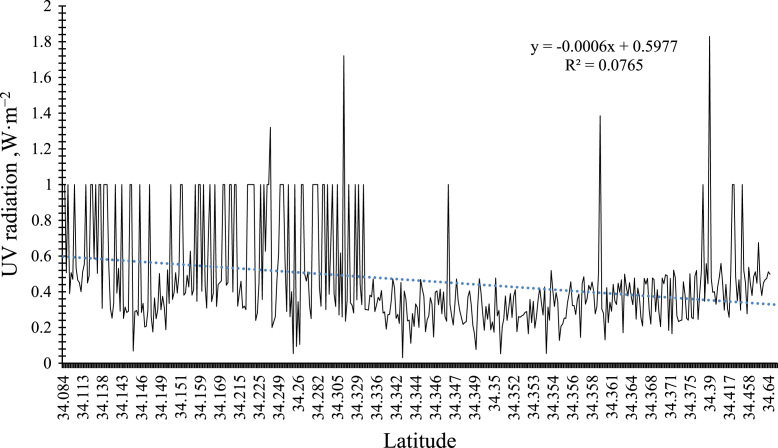
Comparison of the relationship between latitude and UV ray.

Fig. 2 shows that there is a meaningful relationship between latitudinal changes and UV radiation. In other words, as the latitude increases, the intensity of UV radiation decreases (P-value < 0.001).

### 1.3 Relationship between the height above free sea level and the intensity of UV radiation in Mashhad

**Fig. 3:**
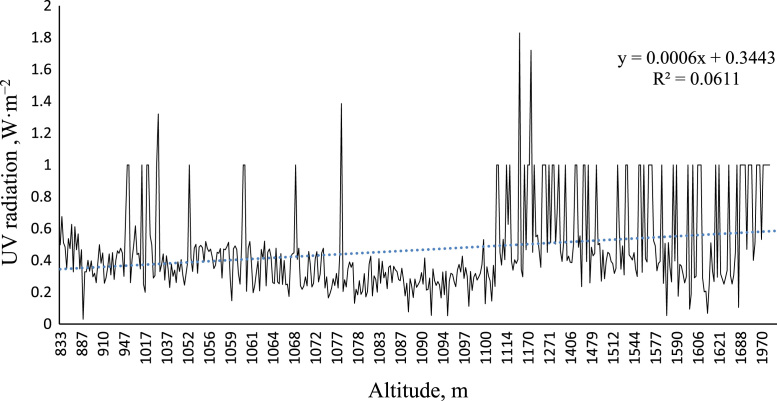
The relationship between the altitude of the open sea with the intensity of UV ray of the Gonabad city.

Fig. 3 show the relationship between the height above sea level and the intensity of UV radiation in Mashhad. As it is shown in Fig. 3 by increasing heights above free sea level, the intensity of UV irradiation significantly increases (P-value <0.001).

### 1.4 The effect of human-made artifacts on the intensity of UV radiation

Table 1 shows the effect of human on the intensity of UV radiation in Mashhad. Table 1 shows that in the village, the average UV was higher than other human-made artifacts, and the lowest level of UV radiation was related to urban artifacts. Table 1 shows that there is a significant relationship between human-made artifacts and UV radiation (P-value < 0.001).

**Table 1:** Effect of manmade effects on ultraviolet radiation intensity in Mashhad.

**Radiation type**	**Average of UV (W m**^**-2**^**)**			
Location	City	Village	Avenue	Other
Manmade effects	0.39	0.63	0.56	0.41

### 1.5 The effect of weather conditions on the intensity of UV radiation in Mashhad

Table 2 shows the effect of climatic conditions on the intensity of UV radiation in Mashhad. Table 2 shows that there is a significant correlation between weather conditions and UV (P-value < 0.001) and the cloudy, semi-cloudy and sunny conditions have the most effect on the intensity of UV radiation, respectively. Also in Table 3, Table 4 meteorological parameters and amount of particulate matter (PM 2.5) in Mashhad (2016–2017) are reported.

**Table 2:** Effect of climatic conditions on the intensity of ultraviolet radiation in Mashhad.

**Radiation type**	**Average of UV (W m**^**-2**^**)**		
Conditions	Cloudy	partly cloudy	Sunny
Climatic effects	0.45	0.66	0.46

**Table 3:** Meteorological parameters, including the number of rainy days and sunny days in Mashhad.

**Parameter**	**Data**
Sunny hours	2857
Rainy days	21
Relative humidity (%)	53
Minimum temperature (°C)	-21
Minimum temperature (°C)	43.8
Average annual temperature (°C)	14.5
Annual average precipitation (mm/year)	233.8
Average annual evaporation (mm/year)	1824
The highest wind speed (m/s)	25

**Table 4:** The amount of particulate matter (PM 2.5) in Mashhad.

**Month**	**Concentration (µg/L)**
January	32.3
February	41.5
March	36.2
April	27.6
May	19.4
June	14.2
July	33.5
August	40.8
September	60.3
October	31.5
November	48.9
December	33.7

## 2 Experimental design, materials and methods

### 2.1 Geographical location of studied are

**Fig. 4:**
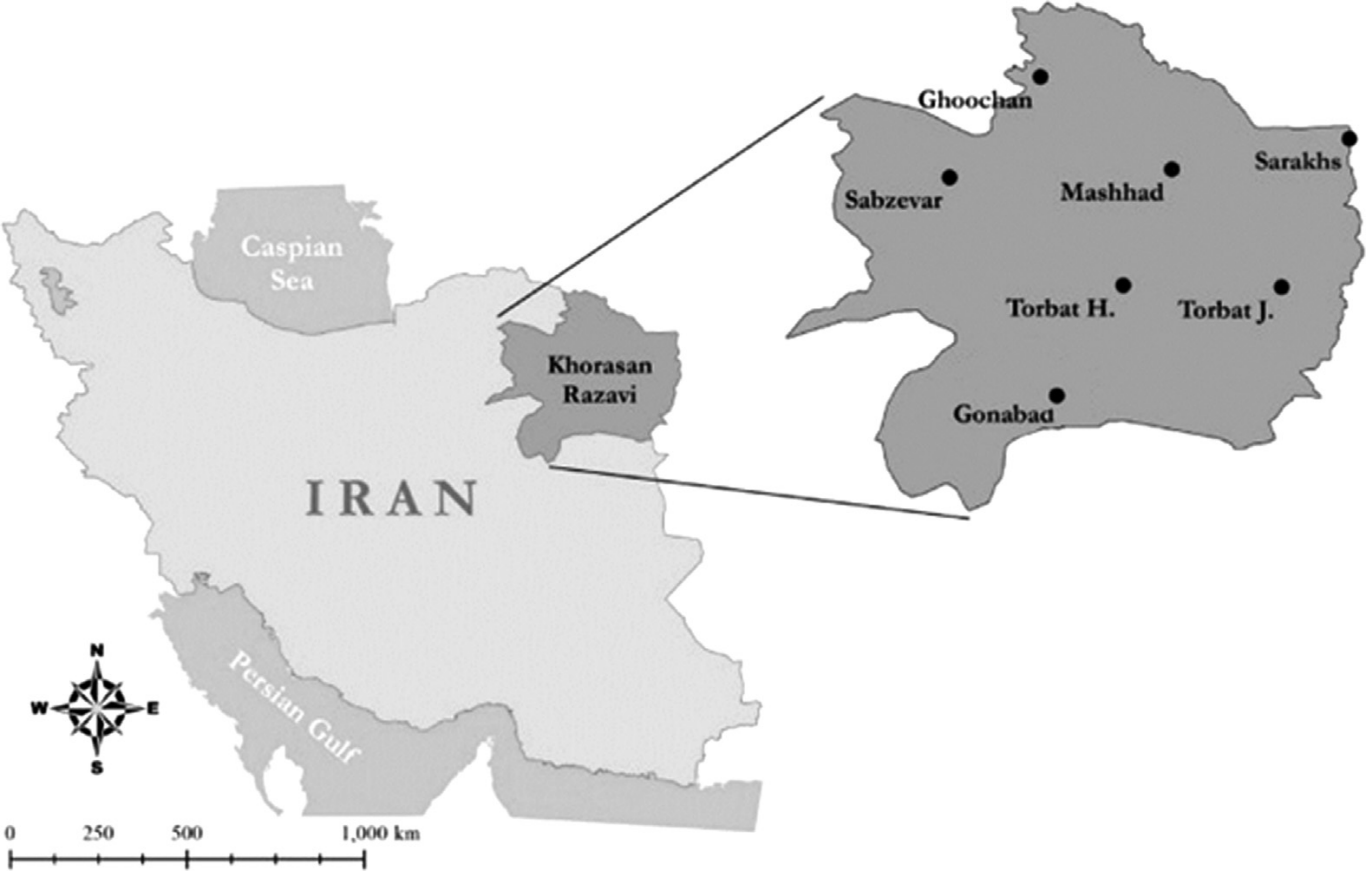
Geographic location of Gonabad city in Iran.

Mashhad city with population over three billion person and an area of about 351 km^2^ is located in 59°,36 E longitude and 36°,18 N latitude (Fig. 4). The neighboring cities are Torbat-e Heydarieh and Ghoochan in the south and north, Sabzevar and Sarakhs in the west. The average height of the city from the open sea level is 1050 m. Its center of Khorasan Razavi province and according to the last divisions of the country.

### 2.2 Method and material

This cross-sectional study was carried out in July of 2017 to determine how the variations of UV radiation were associated with changes in latitude, longitude and, height above the sea level and artificial or human-made and natural artifacts in Mashhad, Khorasan Razavi province in Iran. To this end, the data were collected 2000 sampling stations in different parts of the city (urban, rural, road, etc.). The sampling points were approximately 5000 m far from each other [1]. UV radiation was measured using Hagner radiometer, model UV-A in watt/m^2^ in milliseconds [2]. In this study, all conditions for the selection of sampling points were in accordance with the conditions set out in the valid references [1], [2], [3], [4], [5]. Data were analyzed by SPSS16 software using t-test and ANOVA.

## References

[1] Hokmabadi R.A., Shoja E. (2013). Measurement of cosmic ultraviolet ray intensity (type A) in Bojnurd. J North Khorasan Univ. Med Sci..

[2] Samadi M.T., Golzar Khojasteh B., Rostampour N., Mirazizi L.Shokery (2014). Evaluation of the natural gamma radiation level in residential zones and determination of annual effective exposure dose in the residents of Hamadan province, Iran, 2012. J Kurd. Univ. Med Sci..

[3] Pirsaheb M., Najafi F., Haghparast A., Hemati L., Sharafi K., Kurd N. (2016). The influence of internal wall and floor covering materials and ventilation type on indoor radon and thoron levels in hospitals of Kermanshah, Iran. Iran. Red. Crescent Med. J..

[4] Diffey B.L. (2002). Sources and measurement of ultraviolet radiation. Methods.

[5] Pirsaheb M., Sharafi K., Hemati L., Fazlzadehdavil M. (2015). Radon measurement in drinking water and assessment of average annual effective dose in the west region of Iran. Fresenius Environ. Bull..

