# Supplementary material for: Ultraviolet radiation rate in Mashhad, Iran
Source: Data Brief. 2018 May 25;19:1086–91. doi: 10.1016/j.dib.2018.05.116 (PMC6140285; doi:10.1016/j.dib.2018.05.116)
Supplement: Supplementary file 1 — Supplementary material [file mmc1.doc]

Dear editor(s)

Data in Brief

Greetings!

We would be grateful if you would consider the attached manuscript “**Ultraviolet Radiation Rate in Mashhad, Iran”** for publication in valuable Journal Data in Brief. We declare that this manuscript, or part of it, has neither been published nor is currently under consideration for publication by any other journal. We also declare that we have read the manuscript and approved submission to Journal of Data in Brief. The Project was supported by Mashhad University of Medical Sciences. **None of the authors of this paper have any conflicts of interest to declare.**

Kind Regards,
